# Genetic Characterization and Evolution of H1N1pdm09 after Circulation in a Swine Farm

**DOI:** 10.1155/2014/598732

**Published:** 2014-06-16

**Authors:** Arianna Boni, Gabriele Vaccari, Livia Di Trani, Guendalina Zaccaria, Giovanni Loris Alborali, Davide Lelli, Paolo Cordioli, Ana Maria Moreno

**Affiliations:** ^1^Dipartimento di Sanità Pubblica Veterinaria e Sicurezza Alimentare, Istituto Superiore di Sanità, Viale Regina Elena 299, 00161 Rome, Italy; ^2^Dipartimento di Virologia, Istituto Zooprofilattico Sperimentale della Lombardia e dell'Emilia Romagna, Via A. Bianchi 9, 25124 Brescia, Italy

## Abstract

Following the emergence of the A(H1N1)pdm09 in humans, this novel influenza virus was reverse transmitted from infected people to swine population worldwide. In this study we investigated the molecular evolution of A(H1N1)pdm09 virus identified in pigs reared in a single herd. Nasal swabs taken from pigs showing respiratory distress were tested for influenza type A and A(H1N1)pdm09 by real-time RT-PCR assays. Virus isolation from positive samples was attempted by inoculation of nasal swabs samples into specific pathogen free embryonated chicken eggs (ECE) and complete genome sequencing was performed on virus strains after replication on ECE or from original swab sample. The molecular analysis of hemagglutinin (HA) showed, in four of the swine influenza viruses under study, a unique significant amino acid change, represented by a two-amino acid insertion at the HA receptor binding site. Phylogenetic analysis of HA, neuraminidase, and concatenated internal genes revealed a very similar topology, with viruses under study forming a separate cluster, branching outside the A(H1N1)pdm09 isolates recognized until 2014. The emergence of this new cluster of A(H1N1)pdm09 in swine raises further concerns about whether A(H1N1)pdm09 with new molecular characteristics will become established in pigs and potentially transmitted to humans.

## 1. Introduction

Following the rapid spread of the A(H1N1)pdm09 influenza virus, transmission from infected humans to swine has been detected worldwide [1]. Pigs are susceptible to infection with both avian and human influenza A viruses, playing a crucial role in the emergence of new strains of influenza A viruses (IAVs) against which humans might have little prior immunity. Therefore, swine surveillance represents an important tool for the control of influenza A virus in animals and the further emergence of new influenza strains with pandemic potential. In Italy, swine influenza monitoring programs, based on specific genome detection, virus isolation, and molecular characterization of influenza viruses causing respiratory forms, have been in place since the 1990s. Since April 2009, testing for the A(H1N1)pdm09 on swine samples has also been performed when preliminary screening was positive for influenza type A virus; up to December 2013, twenty-three pandemic strains (Moreno, personal communication) and two reassortant viruses, H1N2 and H1N1 derived from A(H1N1)pdm09, have been detected in more than ten herds located in different Italian regions [2–5].

Considering that a molecular study would provide information on evolution of IAVs in swine, the evolutionary trends of pandemic viruses within a single farm have been examined, through molecular characterization at different time of virus collection.

## 2. Materials and Methods

### 2.1. Influenza Type A Genome Detection and Virus Isolation

Nasal swabs were collected from pigs showing clinical signs and/or lesions related to swine influenza. The samples were tested for influenza A using real-time RT-PCR (RRT-PCR) as previously described by Spackman et al. [6]. Positive samples were further tested for the A(H1N1)pdm09 by RRT-PCR, according to the Centers for Disease Control and Prevention (CDC) procedure [7], and virus isolation was attempted by cell culture infection on Madin-Darby canine kidney and CACO-2 cells and by allantoic sac route infection of 9–11-day-old embryonated chicken eggs (ECE).

Culture supernatant (CS) of tissue culture cells and allantoic fluid (AF) collected from ECE were tested by haemagglutination assay using chicken erythrocytes performed as described in OIE manual 2012 [8] and RRT-PCR assays for influenza type A and A(H1N1)pdm09.

### 2.2. Detection of Swine Respiratory Pathogens

Nasal swabs were cultured using different media for the most common swine respiratory bacterial pathogens. The presence of porcine reproductive and respiratory syndrome virus (PRRSV), porcine circoviruses of type 2 (PCV2), and* Mycoplasma hyopneumoniae* was tested using RT-PCR, multiplex PCR, and PCR assays, respectively, as previously described [10–12].

### 2.3. Genome Sequencing and Phylogenetic Analysis

Full genome sequencing was performed using vRNA extracted from nasal swabs or from ECE virus isolates using QIAamp viral RNA minikit (Qiagen, Germany), following the manufacturer's instructions.

cDNA synthesis and amplification of the entire genome have been obtained with 46 M13 tailed primer pairs representing overlapped genetic fragments of the influenza A genome, as previously described [4], using the SuperScript III One-Step RT-PCR System with Platinum Taq High Fidelity (Invitrogen, Carlsbad, CA, USA).

Sequencing reactions were obtained using the BigDye terminator v3.1 and purified by BigDye XTerminator Purification Kit (Applied Biosystems, Foster City, CA, USA). Sequences were revealed in a 3130 Genetic Analyzer and analysed using the SeqScape software version 2.5.

Multiple sequence alignments were made using ClustalW and phylogenetic trees were created using MEGA 5.2, through the neighbor-joining distance method (Kimura's two-parameter distance model) [13]. Each tree is a consensus of 1000 bootstrap replicates. In order to verify topologies, phylogenetic trees were also constructed by maximum likelihood within PHYML v.3.0 using the HKY85 nucleotide substitution model. Alignments for internal genes were created individually using BioEdit v 7.1.11, manually inspected, and trimmed to include coding regions only. Concatemers of all 6 internal gene segments from Italian swine strains were generated (PB2, PB1, PA, NP, M, and NS) prior to phylogenetic tree construction. Concatemers were similarly generated for reference strains for which the complete genome was retrieved from NCBI influenza virus database.

Predicted glycosylation sites on hemagglutinin (HA) and neuraminidase (NA) of all viruses were performed using NetNGlyc 1.0. A threshold value of 0.5 average potential score was set to predict glycosylated sites.

Putative antigenic sites in the HA gene were identified by alignment to A/Puerto Rico/8/1934 [14, 15].

### 2.4. Nucleotide Sequence Accession Numbers

The following GenBank accession numbers of complete genomes of the five swine influenza viruses (SIVs) (Table 1) were assigned: A/Sw/It/325451/11 (KF823970-KF823977), A/Sw/It/120336/12 (KF309210-KF309217), A/Sw/It/225349-1/12 (KF309218-KF309225), A/Sw/It/225349-2/12 (KF309226-KF309233), and A/Sw/It/225349-4/12 (KF309234-KF309241).

## 3. Results

### 3.1. Virus Isolation and Identification of A(H1N1)pdm09 Viruses

From December 2011 to September 2012, three events of a severe respiratory disease were observed in a farrow-to-finish pig farm in the province of Benevento (South Italy), where the various production phases (mating, gestation, farrowing, nursery, and growing-finishing) were located in separated buildings. The sanitary level at the farm, where 1150 sows were reared, was high, in absence of Aujeszky's disease or PRRS detection and vaccination against swine influenza had not been applied. Respiratory clinical signs as fever, dyspnea, coughing, and anorexia were observed only in weaning pigs; an increase in weaning deaths of 2% over the previous year was also detected. Gross lesions, in most cases, consisted of purple areas of consolidation in apical and cardiac lobes of lungs, interlobular edema, and enlarged mediastinal lymph nodes. Nasal swabs were collected for diagnostic purposes from weaning pigs showing clinical signs and submitted to the diagnostic laboratory.

The first identification of A(H1N1)pdm09 virus infection in pigs was performed in December 2011, by a specific RRT-PCR, without virus isolation after ECE or cell cultures infection; however, sequencing analysis of original virus sample allowed the full genome characterization of the index case A/Sw/It/325451/11 (SIV-1). A second event of viral infection in pigs was detected after five months (May 2012), with successful virus isolation after ECE and cell culture infection of A/Sw/It/120336/12 (SIV-2) virus. Furthermore, in September 2012, a respiratory disease in pigs was reported in the same farm and resulted in identification and isolation on ECE and cell culture of three SIVs: A/Sw/It/225349-1/12 (SIV-3), A/Sw/It/225349-2/12(SIV-4), and A/Sw/It/225349-4/12 (SIV-5). Nasal swabs, AF, and CS of SIVs 2-3-4-5 were tested positive by the specific RRT-PCR against A(H1N1)pdm09.

Differential diagnosis for respiratory pathogens such as bacteria,* M. hyopneumoniae*, PRRSV, or PCV2, which are involved in the porcine respiratory complex, was performed with negative results on all clinical samples.

### 3.2. Genomic Sequencing and Phylogenetic Analysis

Full genome sequencing was obtained from nasal swab of SIV-1 and from the AF isolates of SIVs 2-3-4-5 (Table 1). Gene sequences were compared with influenza sequences available from a public database (Influenza Virus Resource, http://www.ncbi.nlm.nih.gov/genomes/FLU/FLU.html), confirming the RRT-PCR results of subtype identification.

HAs nucleotide identity between SIV-1 and the other four SIVs 2-3-4-5 ranged from 98.83% to 99.01%, while HA gene sequences of SIVs 2-3-4-5 were highly similar, sharing as much as 99.66% nucleotide identities.

**Figure 1:**
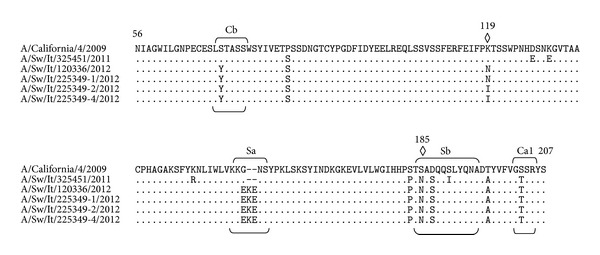
HA1 protein sequence alignment (H3 numbering) of SIVs under study, in comparison with A/California/4/2009 (56aa–207aa). Only amino acid differences from the reference sequence are indicated: differences in the antigenic sites, Sa, Sb, Ca1, and Cb, are underlined. Diamonds indicate new potential glycosylation sites.

HA protein sequence alignment showed that only SIVs 2-3-4-5 contained a unique 2-amino acid (AA) insertion, represented by lysine (K) and glutamic acid (E) at position 155 (Figure 1). Comparison of HA protein sequences with A/California/04/2009 reference virus revealed in SIV-1 a total of 12 AA substitutions: three in Sb antigenic site (S185N, D187S, and S190I) and one in Ca1 antigenic site (S203T) (Table 2(a)). SIVs 2-3-4-5 showed up to 12 AA substitutions, some located within the antigenic sites Sa (G155E), Sb (S185N, D187S), Ca1 (S203T), and Cb (S71Y) (Figure 1 and Table 2(a)).

Since point mutations may cause emergence or loss of Asn-X-Thr/Ser motifs and therefore attachment or loss of N-glycans, leading to alterations in, respectively, the antigenicity or receptor specificity of HA, we analyzed the protein glycosylation sites. All SIVs under study revealed the same S185N substitution in the Sb antigenic site that represents one additional potential glycosylation site; SIVs 2 and 3 showed a further glycosylation site as a consequence of K119N change in the HA stalk (Figure 1).

HA predicted amino acid sequences of all SIVs showed AA changes in a phylogenetically important region (PIR), PIR-I (S183P) and (S185N) [9]; furthermore, the cluster of SIVs with AA insertion showed changes in two different PIRs, PIR-C (S71Y) and PIR-E (K119N/I), whereas only SIV-3 had one AA change in PIR-Q (T474 M) (Table 2(a)).

Sequence analysis of NA protein of all five SIVs showed up to 12 AA differences with A/California/4/09, some on PIRs (Table 2(b)). NA nucleotide identity between SIV-1 and SIVs 2-3-4-5 ranged from 98.27 to 98.42%, whereas nucleotide identities among SIVs 2-3-4-5 ranged from 99.57 to 99.86%. The seven potential glycosylation sites on NA (four on the stalk and three on the head of the glycoprotein) of A(H1N1)pdm09 viruses were confirmed; only SIV-2 showed an additional potential glycosylation site at position 416 at the top of head of NA protein.

Amino acid changes in internal proteins of influenza A viruses can indicate the simulated host range factors; therefore the changes in the AA of PB2, PA, NP, and M2 were also analyzed. A total of 25 nonsynonymous mutations were observed in the internal gene segments (Table 2(c)). Comparison with internal genes of all available A(H1N1)pdm09 sequences showed four unique AA changes in PB2 (K340N), PA (V521L, E538A), and NS1 (I112V), not previously found (Table 2(c)).

All SIVs under study confirmed the presence of PB1-F2 peptide and a PA-X protein-coding region identified in A(H1N1)pdm09 viruses with a total length of 11 and 232 AA, respectively.

**Figure 2:**
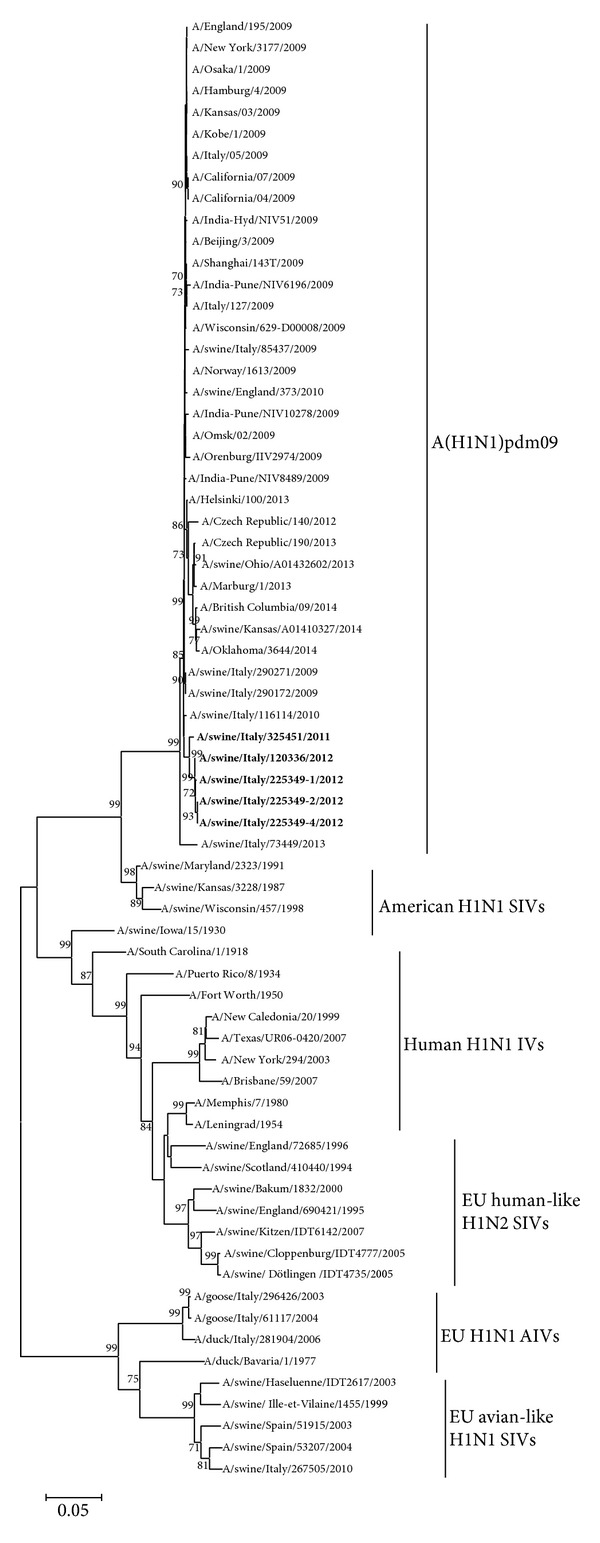
Phylogenetic relationship of the HA gene of SIVs under study with avian (AIVs), swine (SIVs), and human (IVs) origin H1 subtypes. Scale bar indicates nucleotide substitutions per site.

**Figure 3:**
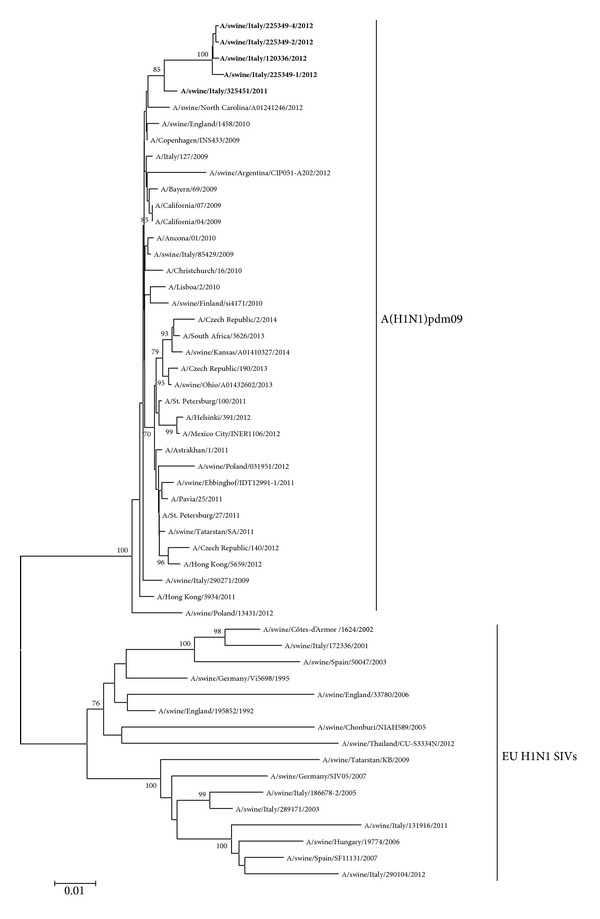
Phylogenetic tree of selected swine and human N1 sequences, obtained from NCBI or GISAID databases. SIVs identified in this study are indicated in bold. Scale bar indicates nucleotide substitutions per site.

Phylogenetic analysis of hemagglutinin and neuraminidase was based on HA and NA sequences of SIVs under study and representative H1 and N1 influenza A viruses of human, avian, and swine origin (Figures 2 and 3).

**Figure 4:**
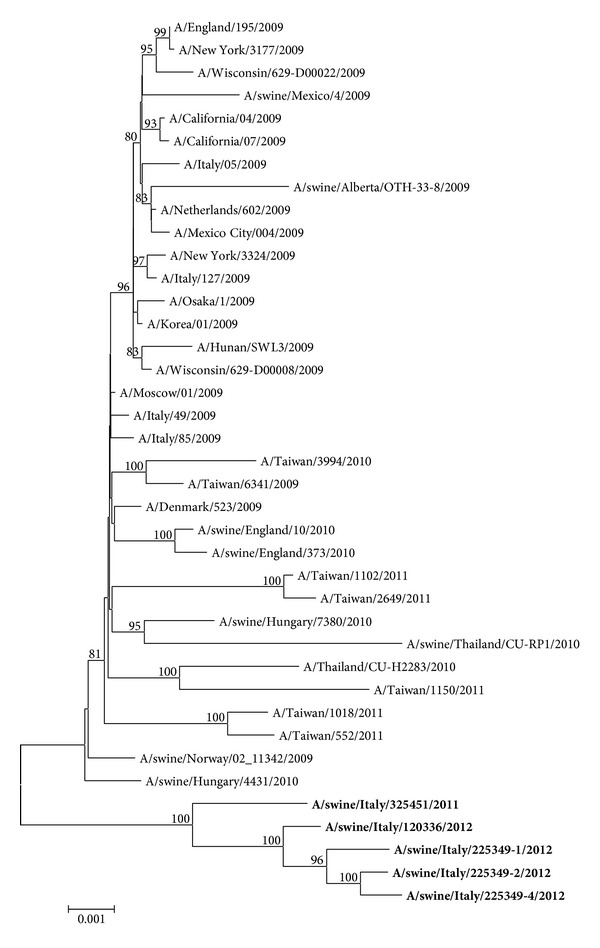
Concatenated internal genes derived phylogenetic tree of A(H1N1)pdm09. Sequences of SIVs under study are indicated in bold. Scale bar indicates nucleotide substitutions per site.

In Figure 4, the phylogenetic tree of concatenated internal gene segment sequences (10028 nts, *n* = 41 seqs) from the SIVs under study and representative A(H1N1)pdm09 influenza viruses collected during 2009–2012 from different geographical regions is represented.

All the phylogenetic trees revealed a very similar topology, with all the five Italian SIVs under study forming a separate cluster, branching outside the A(H1N1)pdm09 isolates detected until 2014, with significant bootstrap values (99.0, 85.0, and 100.0 for the HA, NA, and internal concatenated gene trees, resp.).

## 4. Conclusions

Pigs are considered key intermediate mammal hosts, acting as mixed vessel for genetic reassortment of IAVs; therefore, swine surveillance represents an important tool to control the emergence of potential new pandemic strains. Worldwide detection and circulation of A(H1N1)pdm09 in swine raised concerns about the transmission to humans of strains with different antigenic characteristics, mainly derived from the accumulation of AA substitutions in HA and NA proteins.

To better understand the molecular epidemiology of IAVs in swine, the evolutionary trends of A(H1N1)pdm09 viruses circulating within a single swine farm have been examined, through full genome sequencing and phylogenetic analysis of strains detected during swine surveillance, over a period of nine months. Phylogenetic analysis evidenced that the five SIVs detected have a common origin, suggesting that in a short time the A(H1N1)pdm09 virus after circulation in animals was able to evolve and accumulate significant nucleotide changes, mainly on HA and NA glycoproteins.

Comparison, at protein level, of SIVs individual gene segments with the A(H1N1)pdm09 prototype strain A/California/04/2009 revealed up to 38 AA substitutions scattered throughout the eight gene segments, mainly on the last identified viruses (SIV-4 and SIV-5) and on HA (12 to 13 AA substitutions) and NA (4 to 9 AA substitutions) proteins.

Moreover, HA molecular analysis showed, in four out of five SIVs, a unique significant AA change, represented by 2-AA-insertion at the HA receptor binding site (RBS). The observed AA insertion disrupted the Sa site, one of the five antigenic sites (Sa, Sb, Ca1, Ca2, and Cb) located at the globular head of the hemagglutinin of H1 viruses (Figure 1). Such insertion may derive from slippage of RNA polymerase, since the six inserted nucleotides are an exact repetition of the previous six nucleotides present in this site. Previous studies suggested that the Sa region is an immunodominant antigenic site on the A(H1N1)pdm09 virus, which may drift as the virus evolves [16–18]; therefore observed AA changes, occurring either in the antigenic sites or on the surface of the HA molecule, may have an effect on antibody recognition.

The observation that the AA insertion was not detected in the index case (SIV-1), but it was maintained in all the viruses subsequently identified in the swine herd, might suggest higher virus fitness, possibly due to a virus antibody escape.

Acquisition of additional glycosylation sites in the HA has been also associated with immune evasion mechanisms, as demonstrated with human influenza viruses [19, 20]. This tendency towards the accumulation of glycosylation sites on the globular head of the HA was previously observed as a result of antigenic drift after circulation of the A(H1N1)pdm09 virus [21, 22].

Common to all five SIVs (Table 2(a)), the S185N change determines a further potential glycosylation site in the RBS. Additional potential glycosylation site (K119N) at the head of HA, recently associated with resistance to neutralizing antibodies [23], was observed in SIV-2 and SIV-3. This glycosylation site is however absent in the other two isolates observed at the same time point of SIV-3.

Genetic data of the HA in SIVs confirm the increase of AA changes in the Sb of A(H1N1)pdm09 viruses, as recently reported [24]. Molecular data suggesting antigenic change of viruses will be validated by antigenic analysis in a further study.

Two unique AA changes in NA (Table 2(b)) were also found, (N248G) in all SIVs under study and (I418 M) in SIVs 3-4-5; also internal genes of the five SIVs show AA substitutions not previously found in A(H1N1)pdm09 viruses (PB2 K340N; PA V521L, E538A; NS1 I112V) (Table 2(c)).

All five SIVs possessed the NA H275 residue, a known marker for sensitivity to the neuraminidase inhibitor (oseltamivir) [25] and the genetic marker 31N in the M2 gene, confirming the amantadine resistance of A(H1N1)pdm09 viruses [26]. None of the other listed AA substitutions (Table 2(c)) was previously implicated with replication, virulence, or pathogenicity markers.

The phylogenetic analysis of HA, NA, and internal gene sequences (Figures 2, 3, and 4) showed that SIVs under study form a clearly distinct cluster from the representative A(H1N1)pdm09 sequences identified until now (ECDC, 2014) [27]; such results are suggestive of evolution of the five SIVs under study from a common ancestor virus.

The index case SIV-1 showed throughout its genome, several unique substitutions that were not observed in the subsequent isolates. This feature and the 2-AA-insertion on the HA gene, present exclusively in later isolates, suggest a better fitness to influenza virus replication in swine of the last isolates.

Recently, Detmer et al. [28], in a large study regarding genetic clusters of H1 lineage swine viruses circulating in North America, reported a similar 2-AA-insertion on H1N1 SIVs showing strong binding in the lower respiratory tract of pigs. Therefore further studies should be undertaken to verify the association of such pathogenetic feature to the 2-AA-insertion also in the SIVs 2-3-4-5. Such evidence would be important considering the risk posed to human population by a modified A(H1N1)pdm09 virus, not matching the vaccine virus.

## Figures and Tables

**Table 1:** Data on A(H1N1)pdm09 obtained from swine samples.

Organism	Collection-date	Passage history	Accession numbers
A/Sw/It/325451/2011 (SIV-1)	Dec. 2011	NO	KF823970–KF823977
A/Sw/It/120336/2012 (SIV-2)	May 2012	First passage AF	KF309210–KF309217
A/Sw/It/225349-1/2012 (SIV-3)	Sept. 2012	First passage AF	KF309218–KF309225
A/Sw/It/225349-2/2012 (SIV-4)	Sept. 2012	First passage AF	KF309226–KF309233
A/Sw/It/225349-4/2012 (SIV-5)	Sept. 2012	First passage AF	KF309234–KF309241

**Table:** (a) HA protein

Residue number	71	83	119	127	130	146	155	155 (a, b)	183	185	187	190	197	203	259	449	474	480
A/California/04/09	S	P	K	D	K	K	G	—	S	S	D	S	T	S	R	V	T	K
A/Sw/It/325451/11	—	S	—	E∗	E∗	R∗	—	—	P	N	S∗	I∗	A	T	K∗	—	—	R∗
A/Sw/It/120336/12	Y	S	N	—	—	—	E	KE	P	N	S∗	—	A	T	—	I∗	—	R∗
A/Sw/It/225349-1/12	Y	S	N	—	—	—	E	KE	P	N	S∗	—	A	T	—	I∗	M	R∗
A/Sw/It/225349-2/12	Y	S	I∗	—	—	—	E	KE	P	N	S∗	—	A	T	—	I∗	—	R∗
A/Sw/It/225349-4/12	Y	S	I∗	—	—	—	E	KE	P	N	S∗	—	A	T	—	I∗	—	R∗

	PIRC		PIRE						PIRI	PIRI							PIRQ	

**Table:** (b) NA protein

Residue number	17	20	47	77	106	127	220	248	339	416	418	442
A/California/04/2009	I	A	E	G	V	L	R	N	S	D	I	S
A/Sw/It/325451/11	—	T	—	—	I	—	—	G∗	—	—	—	I
A/Sw/It/120336/12	—	—	G	—	I	S	K	G∗	L	N	T∗	I
A/Sw/It/225349-1/12	—	—	G	—	I	S	K	G∗	L	—	M∗	I
A/Sw/It/225349-2/12	V	—	G	—	I	S	K	G∗	L	—	M∗	I
A/Sw/It/225349-4/12	—	—	G	E	I	S	K	G∗	L	—	M∗	I

	PIRA		PIRB	PIRC				PIRF	PIRJ			

**Table:** (c) Internal gene proteins

Gene segment	PB2			PB1		PA							NP				M2		NS1				NS2	PA-PAX	
Residue number	225	340	676	216	632	201	216	224	379	521	538	628	53	100	186	454	43	65	65	109	112	123	83	199	221
A/California/04/2009	G	K	T	G	V	I	D	P	V	V	E	V	D	V	V	E	T	T	V	Q	I	I	M	R	R
A/Sw/It/325451/11	—	N∗	—	—	A	V	—	S	I	L∗	A∗	L∗	E	I	—	—	—	K∗	—	—	V∗	V	—	K	Q
A/Sw/It/120336/12	—	N∗	—	—	—	V	—	S	I	L∗	A∗	—	E	I	—	D	—	—	—	—	V∗	V	—	K	Q
A/Sw/It/225349-1/12	—	N∗	—	S	—	V	—	S	I	L∗	A∗	—	E	I	—	D	I	—	M	R	V∗	V	I	K	Q
A/Sw/It/225349-2/12	—	N∗	—	—	—	V	—	S	I	L∗	A∗	—	E	I	I	D	—	—	—	—	V∗	V	I	K	Q
A/Sw/It/225349-4/12	S	N∗	A∗	—	—	V	N	S	I	L∗	A∗	—	E	I	—	D	—	—	—	—	V∗	V	—	K	Q
